# Case Report: Mislabeling of an online “alprazolam triazolobenzophenone” pill leads to severe intoxication with the novel synthetic opioid cychlorphine

**DOI:** 10.3389/ftox.2026.1951240

**Published:** 2026-10-21

**Authors:** Annette Zschiesche, Johannes Nadler, Benedikt Pulver, Martin Scheu, Volker Auwärter, Maren Hermanns-Clausen

**Affiliations:** 1 Institute of Forensic Medicine, Forensic Toxicology, Medical Center - University of Freiburg, Faculty of Medicine, University of Freiburg, Freiburg, Germany; 2 Hermann Staudinger Graduate School, University of Freiburg, Freiburg, Germany; 3 Poisons Information Center Freiburg, Department of General Pediatrics, Adolescent Medicine and Neonatology, Faculty of Medicine, Center for Pediatrics, Medical Center – University of Freiburg, University of Freiburg, Freiburg, Germany

**Keywords:** orphines, novel synthetic opioid (NSO), non-fatal intoxication, mislabeling, alprazolam precursor, designer drugs

## Abstract

**Background:**

Mislabeling of illicit drug products poses a serious risk to public health, especially in the rapidly evolving market for new psychoactive substances (NPS). Products sold as benzodiazepines may contain undisclosed novel synthetic opioids (NSOs), exposing opioid-naïve users to life-threatening respiratory depression.

**Case Report:**

A 34-year-old man ingested a pink tablet purchased online and labeled as “alprazolam triazolobenzophenone”, a ring-opened alprazolam prodrug, also known as “alprazafone”. Approximately 45 min after ingestion, he developed snoring and gasping respiration, hypoxemia and deep coma (Glasgow Coma Scale 3). Prehospital bag-valve ventilation and subsequent intubation resulted in prompt stabilization. The patient could be extubated 6 hours later. Contrary to the labeling, neither alprazolam nor its prodrug was detected in the tablet, blood or urine. Instead, the tablet contained approximately 1 mg of *N*-propionitrile chlorphine (cychlorphine), a piperidine benzimidazolone opioid of the emerging “orphine” class. Routine immunoassay screening for opioids and benzodiazepines was negative; identification required targeted LC-MS/MS. Six hours after ingestion, cychlorphine concentrations were 0.46 ng/mL in blood and 5.0 ng/mL in urine-to our knowledge the first human concentrations reported for this substance. *In vitro*, cychlorphine activated the µ-opioid receptor with an EC_50_ of 2.7 nM, i.e. 4.5-fold more potent than fentanyl (EC_50_ = 12.2 nM) within the same assay, with a binding affinity of K_i_ = 1.09 nM (fentanyl: K_i_ = 1.91 nM). The nitazene protonitazepyne was additionally detected in urine but not in blood or serum, indicating earlier intake and terminal elimination at the time of the event.

**Conclusion:**

This case documents cychlorphine as a highly potent NSO capable of causing life-threatening respiratory depression at sub-nanogram-per-millilitre blood concentrations, and illustrates the specific danger of benzodiazepine products adulterated with opioids. No pharmacokinetic data are available for cychlorphine. The concentration, measured 6 hours post-intake, therefore likely underestimates peak exposure. Clinicians should consider NSO exposure in unexplained respiratory depression after NPS use, even where a benzodiazepine was expected. Accessible drug-checking services, take-home naloxone and NSO-capable confirmatory analysis are needed.

## 1 Introduction

Clinical implications of new psychoactive substance (NPS) use still persist and have gained even more relevance in the context of the “opioid crisis” (Humphreys et al., 2022). Novel synthetic opioids (NSOs), including highly potent μ-opioid receptor agonists like fentanyl analogues and emerging non-fentanyl compounds such as nitazenes and orphines, have become a major driver of opioid-related overdose deaths in recent years. Their extreme potency, combined with the fact that they are frequently found to have been added to street heroin without this being declared, a narrow therapeutic index, and the potential need for higher or repeated naloxone dosing makes them particularly dangerous. They can lead to rapid-onset respiratory depression and a high risk of life-threatening and fatal outcomes even at very low doses (EUDA, 2025a; Cozier et al., 2025; Prekupec et al., 2017; Vandeputte et al., 2021; Vandeputte et al., 2022a; Vandeputte et al., 2022b; Edinoff et al., 2023; Giorgetti et al., 2022).

In general, mislabeling of NPS products can lead to severe and unexpected intoxications with high toxicological risks (Hohmann et al., 2014; Monti et al., 2022; Zschiesche et al., 2025), emphasizing the urgent need for awareness and stringent drug checking contributing to harm reduction and prevention of overdoses (Balcaen et al., 2023; Giulini et al., 2023; Friedman and Ciccarone, 2025). This is of particular concern for NSOs, as users expect a pharmacological effect different from an opioid effect and are unaware that the product contains a substance of an entirely different pharmacological class. NSOs have increasingly been declared as benzodiazepines (e.g., alprazolam or diazepam). In June 2024, Ireland reported 20 of such cases where counterfeit benzodiazepine tablets contained the potent nitazene protonitazene (EUDA, 2025b). Such misdeclarations increase the risk of serious opioid overdoses.

Cychlorphine, also referred to as *N*-propionitrile chlorphine (IUPAC: 3-(3-{1-[1-(4-chlorophenyl) ethyl]piperidin-4-yl}-2-oxo-2,3-dihydro-1*H*-1,3-benzimidazol-1-yl) propanenitrile), is a synthetic opioid of the piperidine benzimidazolone class and exhibits structural analogy to chlorphine (see Figure 1). Benzimidazolone opioids (often referred to as ‘orphines’), exemplified by cychlorphine, chlorphine and spirochlorphine, are differentiated from benzimidazole opioids (‘nitazenes’), such as protonitazepyne, by the presence of a ketone group at the 2-position of the benzimidazole ring. In Europe, cychlorphine was first identified in Sweden in December 2024 and in the United States in August 2024 (CSFRE, 2026; 2025; EUDA, 2025c).

**FIGURE 1:**
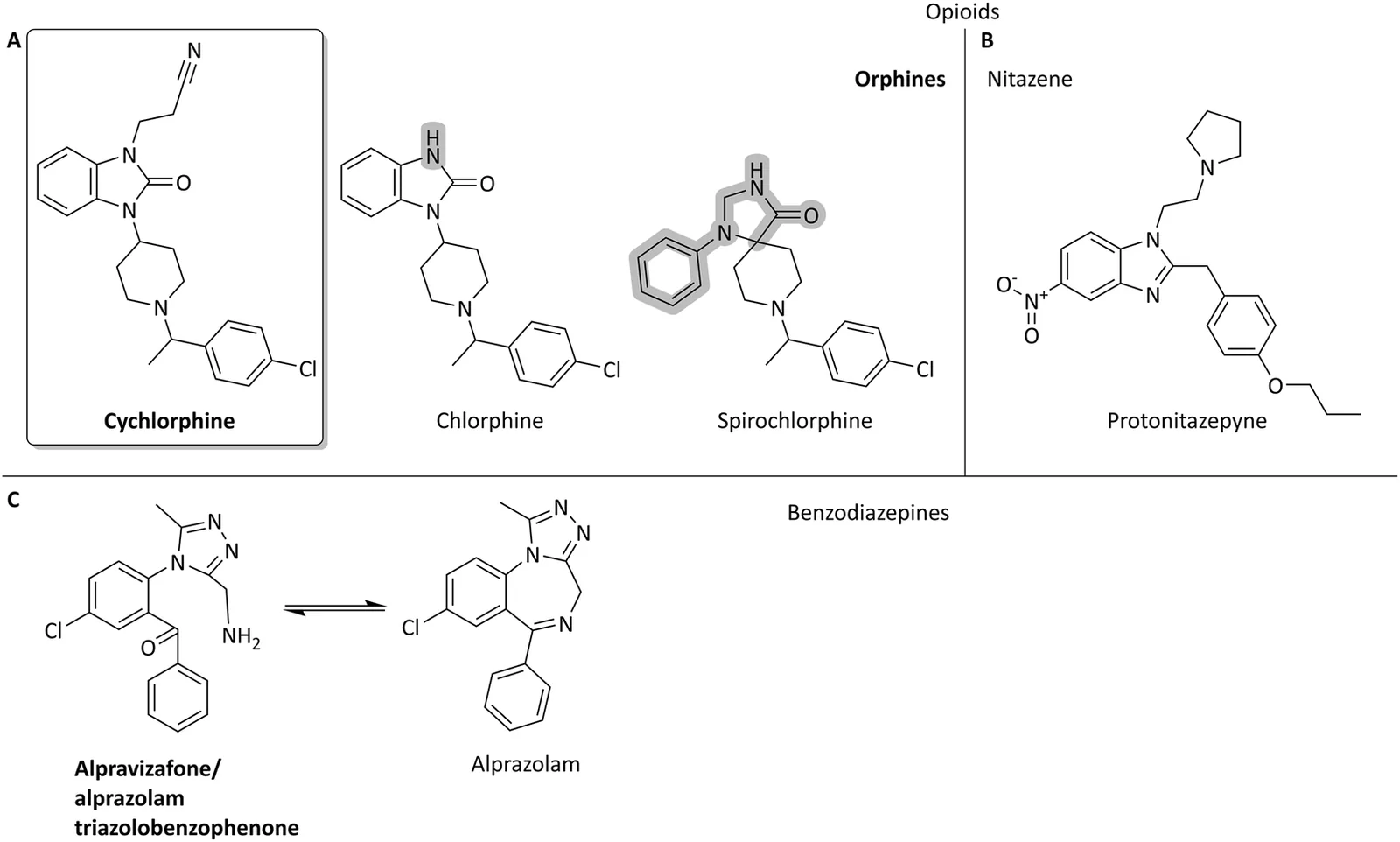
Structures of **(A)** Cychlorpine and two structural derivatives from the “orphine” synthetic opioid group. The structural differences are marked in gray. **(B)** A nitazene representative: protonitazepyne, **(C)** Alprazolam triazolobenzophenone/alprazafone, the ring-opened prodrug of alprazolam.

Ring-opened benzodiazepine prodrugs such as rilmazafone and clonazafone undergo spontaneous or enzymatic cyclization to active rilmazolam and clonazepam, following oral administration (Kronstrand et al., 2023; Malzacher et al., 2025). The seized pills in the presented case here were declared as “alprazolam triazolobenzophenone” (IUPAC: {2-[3-(Aminomethyl)-5-methyl-4*H*-1,2,4-triazol-4-yl]-5-chlorophenyl}(phenyl) methanone), a ring-opened prodrug of the benzodiazepine alprazolam (Cho et al., 1986).

A case reported in 2025 involved a 36-year-old man who insufflated a substance believed to be alprazolam. A toxicological analysis identified xylazine, fentanyl, and predominantly cychlorphine in the powder (Sprague et al., 2025). Another case reported by a drug-checking service in Paris concerned a 20-year-old man who snorted a powder containing 5% cychlorphine (Dugues et al., 2026). Furthermore, a 20-year-old woman was found unresponsive with respiratory depression after taking alprazolam. LC-QToF-MS confirmed cychlorphine use (Kusko et al., 2026). In all instances, blood and urine concentrations were not assessed. The present case adds quantitative blood and urine data, estimated dose, as well as potency and affinity parameters of cychlorphine compared to fentanyl.

## 2 Case report

A 34-year-old male was admitted to the emergency room following apnea and cyanosis. He later reported having ingested one 1 mg tablet of “alprazolam triazolobenzophenone”, a research chemical marketed as an “alprazolam precursor” (Figure 2), at 10:00 p.m. to help him get to sleep. The patient had prior experience with alprazolam purchased online.

**FIGURE 2:**
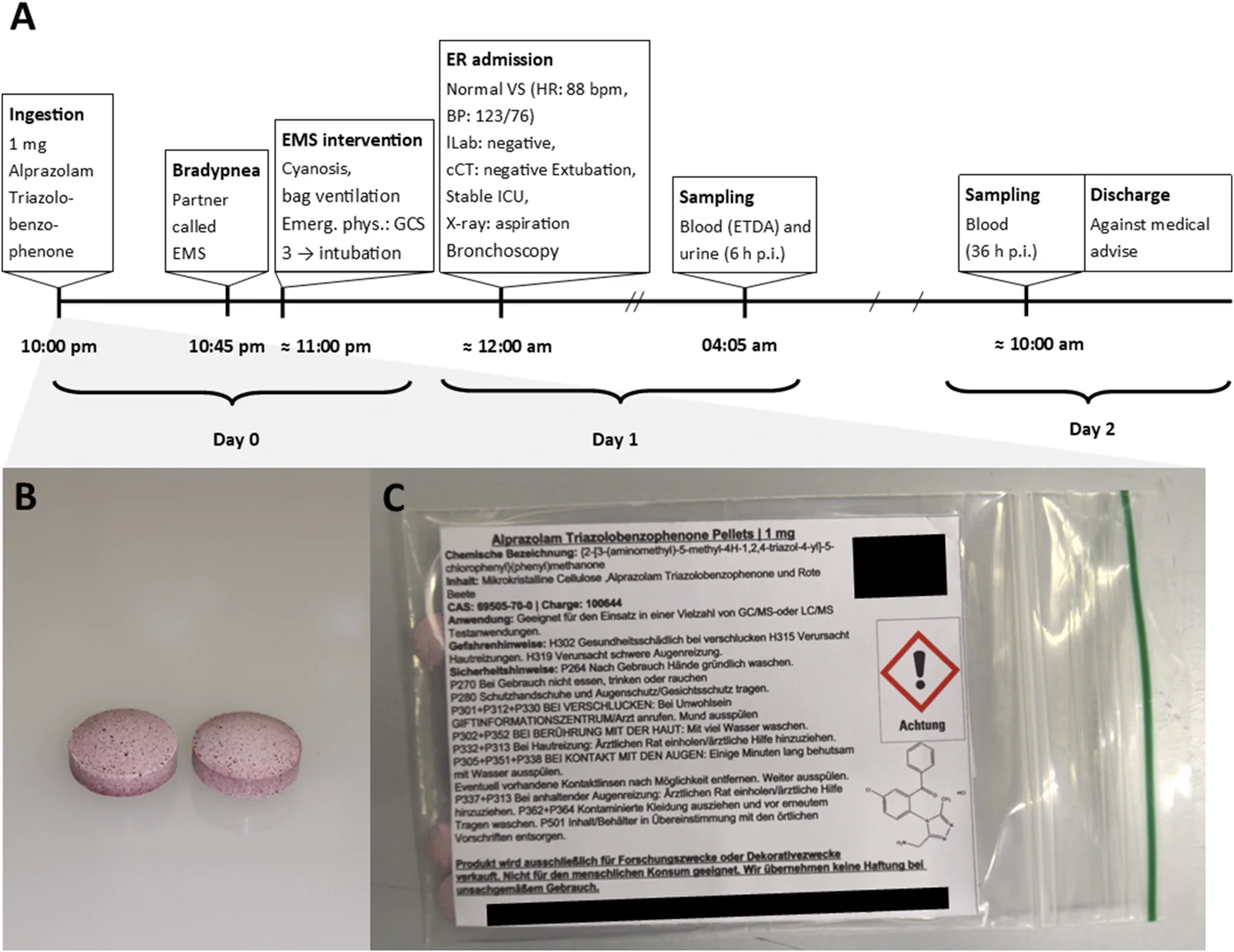
**(A)** Simplified timeline of the patient’s clinical course following ingestion of a tablet declared to contain 1 mg “alprazolam triazolobenzophenone”. EMS, emergency medical services; emerg. phys.: emergency physician; GCS, Glasgow Coma Scale; VS, vital signs; ER, emergency room; cCT, cranial computed tomography; p. i., post-ingestion. **(B)** Two of the three seized pink pills with dark red speckles (Medical Center–University of Freiburg, 2025), **(C)** Three pink pills declared as “alprazolam triazolobenzophenone”, other ingredients: microcrystalline cellulose, red beetroot. Danger and safety notices are attached. Translation into English: “This product is not intended for human consumption and is sold for research or decorative purposes only. No liability is accepted for improper use.”.

According to his partner, he began to snore before developing gasping for breath. A 5-min resuscitation of the cyanotic patient with bag ventilation was performed by a nearby first-responder. The emergency physician, who arrived shortly afterwards, intubated and ventilated the patient after applying midazolam and fentanyl. On admission at the emergency room the patient was ventilated with good oxygen saturation. He was still comatose (GCS 8). Heart rate and blood pressure were within the normal range (88 bpm, 123/76 mmHg). Besides a slightly elevated troponin (20 ng/L) and a blood glucose of 152 mg/dL, electrolytes, kidney and liver function tests as well as blood gases were within the normal range. The patient was ventilated for 6 hours. Naloxone was not applied to the patient at any time, because the intake of an opioid was not suspected. After slowly regaining consciousness, he was extubated on the next day. The patient was discharged against medical advice 2 days after admission. Urine and EDTA-stabilized blood for toxicological analyses were collected 6 h post-ingestion, a second blood sample was obtained after 36 h.

According to the questionnaire, the patient did not expect the pill to contain an NSO. By his own account, he had prior experience with herbal mixtures and various other substances, but not with methamphetamine or opioids. The patient recalled only the shortness of breath and learned about the further course, including his period of unconsciousness, from the treating physician. After stabilization, he was relieved to have survived the acute event and discharged himself against medical advice 2 days after admission.

## 3 Materials and methods

### 3.1 Chemicals

Acetonitrile (ACN, UPLC grade), ammonium formate (10 M) and dimethyl sulfoxide (DMSO) were bought from Sigma-Aldrich (Steinheim, Germany). Deionized water was prepared using a Medica Pro deionizer from ELGA (Celle, Germany). Formic acid (p.a.) and potassium dihydrogen phosphate were purchased from Carl Roth GmbH (Karlsruhe, Germany). Isopropanol (Prepsolv) was obtained from Merck (Darmstadt, Germany). Merck (Darmstadt, Germany) produced the β-glucuronidase/aryl-sulfatase (*Helix pomatia*). MOR membrane and GTP G_i_ binding assay kits (GTP Eu Cryptate reagent, GTP d_2_ antibody, GDP, magnesium chloride, GTPγS, G_i_ protein control and stimulation buffer) were purchased from Revvity (Milan, Italy). UltimaGold™ scintillation liquid, and scintillation vials were procured from Perkin Elmer (Waltham, United States). Tris (hydroxymethyl) aminomethane hydrochloride (TRIS-HCl), bovine serum albumin (BSA) heat shock fraction were purchased from Sigma Aldrich (St. Louis, United States). Polyethylenimine (PEI) was obtained from Thermo Fisher (Schwerte, Germany) and [^3^H]-DAMGO radioligand (250 µCi) was purchased from Revvity (Milan, Italy).

The reference standard *N*-propionitrile chlorphine (hydrochloride), *N*-pyrrolidino protonitazene, nor-orphine, as well as the internal standard (IS) *N*-pyrrolidino protonitazene-d7 (citrate) were purchased from Cayman Chemical (Ann Arbor, Michigan, United States). Fentanyl and fentanyl-d5 were provided by LGC Standards GmbH (Wesel, Germany).

### 3.2 Toxicological analysis

The analyses were performed using in-house, DIN EN ISO/IEC 17025 accredited methods for forensic purposes: gas chromatography-mass spectrometry (GC-MS), liquid chromatography with diode array detection (LC-DAD), immunological screening, targeted liquid chromatography-tandem mass spectrometry (LC-MS/MS) and liquid chromatography-high resolution mass spectrometry (LC-HRMS).

#### 3.2.1 Pill

##### 3.2.1.1 GC-MS

One tablet was extracted with methanol. An aliquot was treated with acidified isopropanol, and derivatized with acetic anhydride/pyridine under microwave irradiation. After addition of deuterated internal standards, samples were analyzed by GC-EI-MS as previously described (Moosmann et al., 2012). Further information can be found in the supporting information.

##### 3.2.1.2 LC-DAD

Ten mg of the pill were weighed three times and extracted with 10 mL methanol each. To 10 µL of sample, 100 µL of eluent was added together with 10 µL of methaqualone (100 μg/mL) as IS. Calibration included 0.1, 0.2, 0.3, 0.5, 0.75 and 1.0 μg/mL of cychlorphine. Injection volume was 10 µL. Content determinations were carried out using LC-DAD in triplicates and were measured twice each. The method can be found in the supporting information.

#### 3.2.2 Urine and blood

Urine and serum samples were analyzed using routine sample preparation protocols. General toxicological screening in urine was conducted by immunoassay screening for drugs of abuse (LSD, benzodiazepines, opiates, amphetamines, cannabinoids, cocaine and its metabolites, as well as 2-ethylidene-1, 5-dimethyl-3, 3-diphenylpyrrolidine (EDDP, a methadone metabolite)). The opiate immunoassay (cobas c OPI2 pack) was calibrated against morphine, with a validated internal cutoff of 21 IA-Units (25 ng/mL morphine after hydrolysis). The benzodiazepine immunoassay (cobas c BENZ II pack) was calibrated against nordiazepam, with a validated internal cutoff of 8 IA-Units (50 ng/mL benzodiazepine equivalent) (Neukamm et al., 2017). Both assays show relevant (> 20%) cross-reactivity with a range of opiates, semisynthetic opioids (Roche Diagnostics GmbH, 2024), and benzodiazepines, including midazolam (121%) and its active metabolite, α-OH-Midazolam (103%) (Roche Diagnostics GmbH, 2021).

In addition, urine was analyzed for NPS by means of targeted LC-MS/MS, encompassing (designer) opioids, metabolites of synthetic cannabinoids, (designer) stimulants, (designer) hallucinogens, and (designer) benzodiazepines as well as LC-QToF-MS. Blood and serum was examined for (designer) opioids, (designer) stimulants (designer) hallucinogens and (designer) benzodiazepines. The methods have been previously described elsewhere (Giorgetti et al., 2024; Grapp et al., 2020; Hermanns-Clausen et al., 2018; Koch et al., 2018; Zschiesche et al., 2026; 2025) and have been updated by incorporating more recent NPS. Urinary creatinine was measured using a cobas 6,000 analyzer with c501 module and Creatinine Jaffé Gen.2 reagent (Roche Diagnostics GmbH, Mannheim, Germany).

For the detection of cychlorphine, 100 µL of the serum samples and EDTA blood sample and 100 µL of the urine sample were used and 10 µL of the IS mixture was added. The urine was enzymatically hydrolyzed by adding 10 µL of β-glucuronidase/arylsulfatase and 100 µL phosphate buffer (0.1 mol/L, pH 6) for 2 h at 45 °C. All samples were then purified by precipitation with ice-cold acetonitrile and ammonium formate. The procedure, the respective LC method and the ion source parameters were adapted and published previously (Malzacher et al., 2025). Since no full in-house validation of the cychlorphine quantification procedure was performed in serum, EDTA blood and hydrolyzed urine, the reported concentrations are considered preliminary estimates and should be interpreted accordingly. The following multiple reaction monitoring (MRM) transitions were used for quantification of cychlorphine: *m/z* 409.2/271.2, 409.2/139.0 and 409.2/84.1 (*t*_R_ = 10.24 min) and fentanyl-d5 as IS (*m/z* 342.2/188.2). For protonitazepyne (*m/z* 409.3/409.3, 409.3/149.1, *t*_R_ = 10.08 min) *N*-pyrrolidino protonitazene-d7 (*m/z* 416.1/98.0) served as IS. External calibration for cychlorphine in urine ranged from 0.5–50 ng/mL and in serum from 0.1–10 ng/mL. For the quantification of protonitazepyne in urine and serum, a calibration ranging from 0.1 to 20 ng/mL was used.

### 3.3 *In vitro* opioid receptor activation and affinity assay

The activation at the µ-opioid receptor (MOR) was evaluated via a HTRF-assay measuring G protein signaling via an increase in fluorescence upon MOR activation. Method details were described previously (Berardinelli et al., 2025). HTRF assays were carried out using receptor membrane preparations from human CHO-K1 cell lines stably expressing the MOR. Test compounds were dissolved in DMSO and a serial dilution ranging from 10^-11^ M to 10^-4^ M was prepared. The assay was performed using at least seven different concentrations of the test compound with the DMSO concentration in the final assay kept constant and below 0.4% (*v/v*) except for the most concentrated solution.

The *in vitro* receptor activation assay was performed in a volume of 20 µL containing 5 µg MOR membrane preparation, the fluorescently labelled GTP analogue, the test compound in different concentrations, and assay buffer. The assay parameters, i.e., basal G protein activation (baseline, measured in the absence of an opioid) and maximal response (specific signal), were assessed. The non-specific signal was determined in the presence of GTPγS. The reaction mixture was incubated overnight to allow optimal FRET signal development. Following incubation, FRET measurements were performed using a time-resolved fluorescence plate reader (VICTOR Nivo - Multimode Plate Reader, Revvity).

Each compound was tested in at least three independent experiments on different days (N = 3 for cychlorphine, and N = 8 for fentanyl), with duplicates included for each concentration within an experiment. Baseline-correction of signals was performed using Microsoft Excel before transferring to GraphPad Prism (Version 10) to normalize the signals to the maximum response of fentanyl. Approximation of sigmoidal concentration-response curves and subsequent calculation of final potency (EC_50_) and efficacy (E_max_; relative to fentanyl) values (log (agonist) versus response, three parameters) were performed with GraphPad Prism. The efficacy was calculated from the sigmoidal concentration-response curve as the span between basal and specific FRET signal at the maximum effective concentration of the ligand tested (basal signal set to 0).

For the affinity at MOR, an assay for synthetic cannabinoids was adapted (Pulver et al., 2023). To this end, 8 µg human MOR membranes were incubated in the competitive binding assay with 2 nM radioligand [^3^H]-DAMGO and the compounds of interest at 10^-6^ to 10^-12^ M (duplicates, N = 4) at a total assay volume of 200 µL. Fentanyl served as reference compound. Radioactivity was measured using a liquid scintillation counter (TriCarb 2100TR, Perkin Elmer).

## 4 Results

### 4.1 Analytical findings

#### 4.1.1 Pill

The seized pill sample labeled as “Alprazolam Triazolobenzophenon” was analyzed using GC-MS and the Cayman Chemical Spectral Library (Ann Arbor, Michigan, United States) (Cayman Chemical, 2025). Cychlorphine was identified and confirmed by measuring a reference standard. After acetylation, acetylated glucose was detected. According to the LC-DAD analysis, the pill contained 0.54% cychlorphine HCl amounting to 0.87 mg in the whole pill.

#### 4.1.2 Urine and blood

Initial immunochemical screening of urine yielded positive results for amphetamines and benzodiazepines. Opioids, cannabinoids, cocaine and metabolites as well as EDDP were negative. Urinary creatinine was 93 mg/dL. Table 1 lists the quantitative results of drugs of abuse and NPS found in the blood and the urine samples by LC-MS/MS. Additional substances detected only qualitatively in urine by general unknown screening can be found in Supplementary Table S1.

**TABLE 1:** Approximate concentrations of drugs detected in blood/serum and urine [ng/mL] by means of LC-MS/MS. Fentanyl and midazolam were administered therapeutically during emergency treatment.

Analyte/Matrix	EDTA-blood*	Serum^#^	Urine*
Cychlorphine	0.46	0.12	5.0
Protonitazepyne	n.d.	n.d.	58
Fentanyl	0.46	n.d.	56
Norfentanyl	< 0.1	n.d.	41
Cathinone	n.d.	n.d.	87
Norephedrine	29	1.1	11,000
Midazolam	18	n.d.	80
α-Hydroxy midazolam	3.1	n.d.	3,700

* Samples taken 6 h post ingestion.

^#^ Sample taken 36 h post ingestion. n.d., not detected.

### 4.2 *In vitro* opioid receptor activation and affinity

The potency (EC_50_), maximal efficacy (E_max_), and binding affinity (K_i_) of the tested compounds at the µ-opioid receptor (MOR) are summarized in Table 2. EC_50_ and E_max_ data have additionally been included in a report submitted to the EUDA (Vandeputte et al., 2026) and are available upon reasonable request. Data for fentanyl and protonitazepyne were included for comparison from previously published work (Berardinelli et al., 2026).

**TABLE 2:** Functional potency (EC_50_, half-maximal effective concentration) and binding affinity (K_i_, inhibition constant) at the µ-opioid receptor (MOR). E_max_, maximal efficacy relative to fentanyl (100%). Data shown as mean with 95% confidence intervals (95% CI).

Compound	EC_50_ [nM]	95% CI	E_max_ (% fentanyl)	95% CI	K_i_ [nM]	95% CI
Cychlorphine	2.7	1.52–4.78	87.1	79.6–94.7	1.09	0.65–1.81
Fentanyl	12.2	10.1–14.8	99.4	96.6–102	1.91	1.36–2.72
Protonitazepyne*	3.7	2.2–8.7	154	152–186	*0.68*	​
Fentanyl*	25.6	10.1–70.2	100	98–119	*3.62*	​

* Data from (Berardinelli et al., 2026), *Italic*: K_i_ predicted *in silico*.

## 5 Discussion

### 5.1 Clinical finding

The clinical presentation – snoring, bradypnea, gasping, cyanosis, and deep coma–is characteristic of (designer) opioid overdose (Shafi et al., 2022; Baldo and Rose, 2022; Regina et al., 2026; Wilde et al., 2020). Although an initial examination of the pupils would have been clinically informative, the emergency responders did not document the pupil size. Due to the patient’s life-threatening hypoventilation, immediate airway management and ventilation were prioritized in accordance with current guidelines for resuscitation in cases of poisoning (Lavonas et al., 2023).

Compared to a prior cychlorphine case, our patient presented with normal vital signs (heart rate 88 beats/min) on ED arrival despite preceding bradypnea and cyanosis. A 36-year-old patient reported by Sprague et al. (2025) was found unconscious (GCS 3) and apneic on the bathroom floor, displaying bradycardia (53 beats/min) and hypothermia (36.3 °C) after snorting a powder containing cychlorphine. He improved after naloxone administration (4 mg followed by another 1 mg). This suggests shared opioid features including respiratory depression.

### 5.2 Analytical findings

The uptake of alprazafone should lead to the detection of alprazolam in urine and blood; however, the latter was not detected. Instead, cychlorphine was detected in all three biomatrices, with concentrations of 0.46 ng/mL in EDTA blood, and 5.0 ng/mL in urine 6 h post-intake. In the serum sample taken 36 h after ingestion, 0.12 ng/mL were detected. The significantly higher urine concentration and relatively low blood levels indicate advanced renal elimination with systemic exposure still present. The immunoassay for opioids failed to detect the NSO as shown with test strips for a variety of nitazenes (De Vrieze et al., 2024a; Marland et al., 2025). Notably, the cychlorphine concentration in EDTA blood is similar to the fentanyl level (both measured at 0.46 ng/mL). The fentanyl concentration can be classified as therapeutic (Schulz et al., 2020). Neither in urine nor blood/serum, ‘nor-orphine’ (1,3-dihydro-1-(4-piperidinyl)-2*H*-benzimidazol-2-one), a theoretical *N*-desalkyl metabolite of cychlorphine was detected with LC-MS/MS. For comparison, cychlorphine concentrations from our in-house data (October 2025 – March 2026, N = 8 cases) varied markedly across cases and matrices, with blood, serum, and plasma concentrations ranging from 0.1–18.0 ng/mL (median: 2.0 ng/mL, mean 3.7 ng/mL, N = 8) and urine concentrations with 0.1, 10 and 12 ng/mL, indicating, e.g., varying dose, time of last consumption and mode of consumption. Cychlorphine itself was already detected in wasterwater samples in Tennessee (Subedi et al., 2026).

Protonitazepyne was detected exclusively in urine (approximately 58 ng/mL), but not in blood, serum or the pill. This pattern is consistent with intake at some point prior to the index event, from a source other than the tablet, with the substance already close to the terminal elimination phase at the time of blood sampling. No *in vivo* pharmacokinetic data, including half-life, have been published for protonitazepyne. For context, according to De Vrieze et al. (2024b), concentrations of protonitazepyne in blood ranged from 0.3 – 55 ng/mL (median: 1.2 ng/mL, mean: 8 ng/mL; N = 26; predominantly *post mortem*). In a further case, serum concentrations of protonitazepyne of 5.9 ng/mL (*ante mortem*) and 7.9 ng/mL (*post mortem*), together with 8.8 ng/mL in *post mortem* urine (creatinine: 200 mg/dL) were reported (Wrbas et al., 2025). It should be noted that protonitazepyne is a highly potent µ-opioid receptor full agonist, so that a contribution at concentrations below the LOQ of the present method cannot be excluded with certainty. Taken together, however, the acute clinical presentation is more plausibly attributable to cychlorphine than to protonitazepyne.

Norephedrine was detected in EDTA blood (29 ng/mL) and in serum (1.1 ng/mL), with a markedly higher concentration in urine (approximately 11,000 ng/mL). Cathinone was found in urine only (87 ng/mL, i.e., < 1% of the corresponding norephedrine concentration). These findings are consistent with exposure to cafedrine and/or norephedrine. Cafedrine, a norephedrine-theophylline conjugate (Akrinor) widely used in emergency and anesthetic practice for hypotension, releases norephedrine *in vivo*. The minor amount of cathinone is best explained by partial oxidation of norephedrine rather than by cathinone intake – as described before (Schwelm et al., 2020). Midazolam and its primary metabolite, α-hydroxy-midazolam and fentanyl/norfentanyl were present in blood and urine at concentrations consistent with therapeutic use, e.g., for emergency medical sedation (Schulz et al., 2020). The exact doses of fentanyl and midazolam administered during emergency intubation could not be retrieved from the available documentation and were therefore not patient-specific. Both were administered according to standard practice (fentanyl 50–100 μg, midazolam 0.5–1 mg) as part of emergency intubation and ventilation to stabilize the patient for transport to the emergency department.

### 5.3 *In Vitro* opioid receptor activation and affinity

*In vitro* characterization of µ-opioid receptor activation using a modified HTRF-based GTP G_i_ binding assay found cychlorphine to be 4.5-fold more potent than fentanyl (EC_50_ = 2.7 nM vs. EC_50_ = 12.2 nM for fentanyl). Cychlorphine exhibited a higher affinity for the µ-opioid receptor than fentanyl, as indicated by a lower K_i_ (1.09 nM vs. 1.91 nM). For the parent compound chlorphine, EC_50_ values of 16.1 nM (MOR-βarr2 recruitment, E_max_ = 161% relative to fentanyl) and of 126 nM (MOR-mini-G_αi_ recruitment, E_max_ = 352% (fentanyl)) were found. MOR affinity was determined with K_i_ = 1.92 nM exceeding that of fentanyl (K_i_ = 6.29 nM) (Vandeputte et al., 2024). In comparison, the nitazene protonitazepyne is approximately 6.9-fold more potent than fentanyl.

It should be emphasised that EC_50_ and K_i_ values obtained in different assay systems and laboratories are not directly comparable – as illustrated by the fentanyl reference values cited above, which differ by a factor of more than three. Relative potencies are therefore only meaningful within a single assay. Furthermore, *in vitro* potency does not translate directly into *in vivo* effect.

## 6 Conclusion

This case illustrates the clinical and forensic risks arising from mislabeled NPS products. A tablet purchased online as a prodrug of alprazolam contained no benzodiazepine at all, but the highly potent piperidine benzimidazolone opioid cychlorphine at 0.87 mg per tablet. Tablets of identical appearance were offered as “alprazafone” and “alpravizafone” by at least two online vendors. Visual identity does not, of course, establish identical composition, and the actual content of such products cannot be predicted.

Pharmacologically, purchasers expect a benzodiazepine, so that a dose intended as a sedative may cause life-threatening respiratory depression. Analytically, the substitution is invisible to routine screening, since opioid immunoassays do not cross-react with benzimidazolone opioids. Although ring-opened benzodiazepine prodrugs lack the core benzodiazepine structure and would not be expected to directly cross-react with the immunoassay, their rapid conversion to pharmacologically active benzodiazepine-type metabolites, combined with the broad cross-reactivity of the assay, makes immunoassay detection plausible following administration of such prodrugs as well.

For clinical practice, novel synthetic opioids should be considered in any case of unexplained respiratory depression following NPS use, irrespective of the substance the patient believes they have taken. From a public health perspective, accessible drug-checking services and take-home naloxone are the most direct preventive measures for this specific risk constellation.

## Data Availability

The original contributions presented in the study are included in the article/Supplementary Material, further inquiries can be directed to the corresponding author.

## References

[B1] Balcaen M. Ventura M. Gil C. Luf A. Martins D. Cunha M. (2023). Challenges in drug surveillance: strengthening the analysis of new psychoactive substances by harmonizing drug checking services in proficiency testing. Int. J. Environ. Res. Public. Health 20, 4628. 10.3390/ijerph20054628 36901637 PMC10002093

[B2] Baldo B. A. Rose M. A. (2022). Mechanisms of opioid-induced respiratory depression. Arch. Toxicol. 96, 2247–2260. 10.1007/s00204-022-03300-7 35471232

[B3] Berardinelli D. Kutzler J. Taoussi O. Zaami S. Pichini S. Basile G. (2025). Dipyanone, a new methadone-like synthetic opioid: *in vitro* and *in vivo* human metabolism and pharmacological profiling. Arch. Toxicol. 99, 2339–2353. 10.1007/s00204-025-04023-1 PMC12185669 40295323

[B4] Berardinelli D. Taoussi O. Ovat D. Y. Pichini S. Pulver B. Auwärter V. (2026). Human metabolism and pharmacological profiling of protonitazepyne and metonitazepyne, two highly potent nitazenes: prediction of main metabolite activity based on µ-opioid receptor docking simulations. Arch. Toxicol. 100, 543–556. 10.1007/s00204-025-04163-4 PMC12886327 41168532

[B5] Cayman Chemical (2025). Forensic science products cayman. Chemical. Available online at: https://www.caymanchem.com/forensics/publications/csl?srsltid=AfmBOopciWI6q736uVIMoqmN8YBXilsT0JS6JcaxG8yjUlIE08urewxL (Accessed February 19, 2026).

[B6] Cho M. J. Sethy V. H. Haynes L. C. (1986). Sequentially labile water-soluble prodrugs of alprazolam. J. Med. Chem. 29, 1346–1350. 10.1021/jm00158a004 3016261

[B7] Cozier G. Gardner M. Power A. Millea M. Barrett S. Jones G. (2025). The changing landscape of opioid use; strength, adulteration and implications for harm reduction. ChemRxiv. 10.26434/chemrxiv-2025-ltl1w

[B8] CSFRE (2025). Monographs: N-propionitrile chlorphine. Available online at: https://www.cfsre.org/nps-discovery/monographs/n-propionitrile-chlorphine (Accessed August 2, 26).

[B9] CSFRE (2026). Public alert: increase in fatal overdoses linked to novel synthetic opioid N-Propionitrile chlorphine (cychlorphine). Available online at: https://nij.ojp.gov/library/publications/increase-fatal-overdoses-linked-novel-synthetic-opioid-n-propionitrile (Accessed August 2, 26).

[B10] De Vrieze L. M. Stove C. P. Vandeputte M. M. (2024a). Nitazene test strips: a laboratory evaluation. Harm. Reduct. J. 21, 159. 10.1186/s12954-024-01078-8 PMC11350930 39198843

[B11] De Vrieze L. M. Walton S. E. Pottie E. Papsun D. Logan B. K. Krotulski A. J. (2024b). *In vitro* structure–activity relationships and forensic case series of emerging 2-benzylbenzimidazole ‘nitazene’ opioids. Arch. Toxicol. 98, 2999–3018. 10.1007/s00204-024-03774-7 38877156 PMC11324687

[B12] Dugues P. Robin T. Bellouard M. Chenorhokian S. Triguel M. Pfau G. (2026). Multi-analytical identification of the synthetic opioids cychlorphine and methiodone (IC-26) in drug seizures: first detection in Europe. Clin. Chem. Lab. Med. CCLM. 64, 1413–1424. 10.1515/cclm-2025-1428 41507112

[B13] Edinoff A. N. Martinez Garza D. Vining S. P. Vasterling M. E. Jackson E. D. Murnane K. S. (2023). New synthetic opioids: clinical considerations and dangers. Pain Ther. 12, 399–421. 10.1007/s40122-023-00481-6 36826742 PMC9950705

[B14] EUDA (2025a). Threat assessment report: assessing the threat posed by the increased availability, use and harms of highly potent synthetic opioids in the Baltic region. Available online at: https://www.euda.europa.eu/publications/threat-assessment/threat-assessment-report-synthetic-opioids-baltic-region_en (Accessed October 7, 26).

[B15] EUDA (2025b). New psychoactive substances – the current situation in Europe (European drug report 2025). Available online at: https://www.euda.europa.eu/publications/european-drug-report/2025/new-psychoactive-substances_en (Accessed June 29, 2026).

[B16] EUDA (2025c). EDND: Formal Notification Cychlorphine. European Union Drugs Agency. Available online at: https://ednd2.emcdda.europa.eu/ednd/substanceProfiles/1574 (Accessed July 2, 26).

[B17] Friedman J. Ciccarone D. (2025). The public health risks of counterfeit pills. Lancet Public Health 10, e58–e62. 10.1016/S2468-2667(24)00273-1 39755415 PMC12101522

[B18] Giorgetti A. Brunetti P. Pelotti S. Auwärter V. (2022). Detection of AP-237 and synthetic cannabinoids on an infused letter sent to a German prisoner. Drug Test. Anal. 14, 1779–1784. 10.1002/dta.3351 35918775 PMC9804899

[B19] Giorgetti A. Große Perdekamp M. Franchetti G. Pircher R. Pollak S. Pelotti S. (2024). Intoxications involving methoxyacetylfentanyl and U-47700: a study of 3 polydrug fatalities. Int. J. Leg. Med. 138, 1801–1811. 10.1007/s00414-024-03263-7 PMC11306479 38831139

[B20] Giulini F. Keenan E. Killeen N. Ivers J.-H. (2023). A systematized review of drug-checking and related considerations for implementation as A harm reduction intervention. J. Psychoact. Drugs 55, 85–93. 10.1080/02791072.2022.2028203 35060837

[B21] Grapp M. Kaufmann C. Schwelm H. M. Neukamm M. A. Blaschke S. Eidizadeh A. (2020). Intoxication cases associated with the novel designer drug 3′,4′-methylenedioxy-α-pyrrolidinohexanophenone and studies on its human metabolism using high-resolution mass spectrometry. Drug Test. Anal. 12, 1320–1335. 10.1002/dta.2869 32476242

[B22] Hermanns-Clausen M. Müller D. Kithinji J. Angerer V. Franz F. Eyer F. (2018). Acute side effects after consumption of the new synthetic cannabinoids AB-CHMINACA and MDMB-CHMICA. Clin. Toxicol. 56, 404–411. 10.1080/15563650.2017.1393082 29072524

[B23] Hohmann N. Mikus G. Czock D. (2014). Effects and risks associated with novel psychoactive substances: mislabeling and sale as bath salts, spice, and research chemicals. Dtsch. Arzteblatt Int. 111, 139–147. 10.3238/arztebl.2014.0139 24661585 PMC3965957

[B24] Humphreys K. Shover C. L. Andrews C. M. Bohnert A. S. B. Brandeau M. L. Caulkins J. P. (2022). Responding to the opioid crisis in North America and beyond: recommendations of the stanford–*lancet* commission. Lancet 399, 555–604. 10.1016/S0140-6736(21)02252-2 35122753 PMC9261968

[B25] Koch K. Auwärter V. Hermanns-Clausen M. Wilde M. Neukamm M. A. (2018). Mixed intoxication by the synthetic opioid U-47700 and the benzodiazepine flubromazepam with lethal outcome: pharmacokinetic data. Drug Test. Anal. 10, 1336–1341. 10.1002/dta.2391 29637722

[B26] Kronstrand R. Roman M. Johansson A. Wu X. Green H. Truver M. T. (2023). Rilmazafone: a designer benzodiazepine pro-drug involved in fatal intoxications. J. Anal. Toxicol. 47, 640–643. 10.1093/jat/bkad041 37348041

[B27] Kusko R. Liss D. House S. L. House S. B. Krotulski A. Aldy K. (2026). An emerging synthetic opioid in the drug supply: a case of opioid overdose with biological confirmation of cychlorphine. J. Med. Toxicol. 22, 490–492. 10.1007/s13181-026-01146-4 42481906 PMC13633184

[B28] Lavonas E. J. Akpunonu P. D. Arens A. M. Babu K. M. Cao D. Hoffman R. S. (2023). American heart association focused update on the management of patients with cardiac arrest or life-threatening toxicity due to poisoning: an update to the American heart association guidelines for cardiopulmonary resuscitation and emergency cardiovascular care. Circulation 148, e149–e184. 10.1161/cir.0000000000001161 37721023

[B29] Malzacher J. Pulver B. Heller N. Brockbals L. Bolliger S. A. Kraemer T. (2025). Fatal intoxication involving amphetamine, clonazafone, a benzodiazepine prodrug, and fluoro-etonitazene, a new synthetic opioid. Int. J. Leg. Med. 140, 769–778. 10.1007/s00414-025-03627-7 41114844 PMC12956958

[B30] Marland V. Nisbet L. Nic Daéid N. (2025). Evaluation of nitazene immunoassay test strips for rapid *in-situ* detection of nitazene and nitazene analogs in illicit drug samples. Harm. Reduct. J. 22, 137. 10.1186/s12954-025-01287-9 40783738 PMC12335075

[B31] Medical Center – University of Freiburg (2025). Warnung Vor Falsch Deklarierten Tabletten – Lebensbedrohliche Vergiftungen Möglich (Warning About Falsely Labelled Tablets – life-threatening Poisoning Possible).

[B32] Monti M. C. Zeugin J. Koch K. Milenkovic N. Scheurer E. Mercer-Chalmers-Bender K. (2022). Adulteration of low-delta-9-tetrahydrocannabinol products with synthetic cannabinoids: results from drug checking services. Drug Test. Anal. 14, 1026–1039. 10.1002/dta.3220 34997693 PMC9305195

[B33] Moosmann B. Kneisel S. Girreser U. Brecht V. Westphal F. Auwärter V. (2012). Separation and structural characterization of the synthetic cannabinoids JWH-412 and 1-[(5-fluoropentyl)-1H-indol-3yl]-(4-methylnaphthalen-1-yl)methanone using GC–MS, NMR analysis and a flash chromatography system. Forensic Sci. Int. 220, e17–e22. 10.1016/j.forsciint.2011.12.010 22264627

[B34] Neukamm M. A. Bahrami A. Auwärter V. Mehne F. M. P. Höss E. (2017). Evaluation of KIMS immunoassays on a cobas c 501 analyzer for drugs of abuse and ethyl glucuronide testing in urine for forensic abstinence control. Drug Test. Anal. 9, 1217–1223. 10.1002/dta.2154 28024169

[B35] Prekupec M. P. Mansky P. A. Baumann M. H. (2017). Misuse of novel synthetic opioids: a deadly new trend. J. Addict. Med. 11, 256–265. 10.1097/ADM.0000000000000324 28590391 PMC5537029

[B36] Pulver B. Riedel J. Schönberger T. Halter S. Lucas T. Opatz T. (2023). Pharmacology, prevalence in Germany, and analytical data of cyclobutylmethyl- and norbornylmethyl-type synthetic cannabinoid receptor agonists. Drug Test. Anal. 15, 408–425. 10.1002/dta.3427 36541839

[B37] Regina A. C. Goyal A. Mechanic O. J. (2026). Opioid Toxicity, In: Statpearls. Treasure Island (FL): StatPearls Publishing. 29262202

[B38] Roche Diagnostics GmbH (2021). Cobas C Pack BNZ2 – ONLINE DAT Benzodiazepines II: Method Sheet. Mannheim, Germany: Roche Diagnostics GmbH.

[B39] Roche Diagnostics GmbH (2024). Cobas C Pack OPI2 – ONLINE DAT Opiates II: Method Sheet. Mannheim, Germany: Roche Diagnostics GmbH.

[B40] Schulz M. Schmoldt A. Andresen-Streichert H. Iwersen-Bergmann S. (2020). Revisited: therapeutic and toxic blood concentrations of more than 1100 drugs and other xenobiotics. Crit. Care 24, 195. 10.1186/s13054-020-02915-5 32375836 PMC7201985

[B41] Schwelm H. M. Grumann C. Auwärter V. Neukamm M. A. (2020). Application of a chiral high-performance liquid chromatography-tandem mass spectrometry method for the determination of 13 related amphetamine-type stimulants to forensic samples: interpretative hypotheses. Drug Test. Anal. 12, 1354–1365. 10.1002/dta.2886 32589765

[B42] Shafi A. Berry A. J. Sumnall H. Wood D. M. Tracy D. K. (2022). Synthetic opioids: a review and clinical update. Ther. Adv. Psychopharmacol. 12, 20451253221139616. 10.1177/20451253221139616 36532866 PMC9747888

[B43] Sprague J. E. Toms J. A. Ratermann C. F. (2025). Non-fatal opioid overdose associated predominantly with the benzimidazolone, cychlorphine. Clin. Toxicol. 0, 1–2. 10.1080/15563650.2025.2594070 41347459

[B44] Subedi B. Sapkota R. Phillips D. H. (2026). Early warning from wastewater: detection of emerging opioid cychlorphine in rural Tennessee. Environ. Sci. Technol. Lett. 13, 1064–1070. 10.1021/acs.estlett.6c00529

[B45] Vandeputte M. Van Uytfanghe K. Layle N. K. St. Germaine D. M. Iula D. M. Stove C. P. (2021). Synthesis, chemical characterization, and μ-Opioid receptor activity assessment of the emerging group of “nitazene” 2-benzylbenzimidazole synthetic opioids. ACS Chem. Neurosci. 12, 1241–1251. 10.1021/acschemneuro.1c00064 33759494

[B46] Vandeputte M. Krotulski A. J. Papsun D. M. Logan B. K. Stove C. P. (2022a). The rise and fall of isotonitazene and brorphine: two recent stars in the synthetic opioid firmament. J. Anal. Toxicol. 46, 115–121. 10.1093/jat/bkab082 34233349

[B47] Vandeputte M. Krotulski A. J. Walther D. Glatfelter G. C. Papsun D. Walton S. E. (2022b). Pharmacological evaluation and forensic case series of N-pyrrolidino etonitazene (etonitazepyne), a newly emerging 2-benzylbenzimidazole ‘nitazene’ synthetic opioid. Arch. Toxicol. 96, 1845–1863. 10.1007/s00204-022-03276-4 PMC12964052 35477798

[B48] Vandeputte M. Bilel S. Tirri M. Corli G. Bassi M. Layle N. K. (2024). Elucidating the harm potential of brorphine analogues as new synthetic opioids: synthesis*, in vitro*, and *in vivo* characterization. Neuropharmacology 260, 110113. 10.1016/j.neuropharm.2024.110113 39154855

[B49] Vandeputte M. M. Pulver B. Persson M. Bilel S. Lambrecht I. M. Marti M. (2026). Report of the Pharmacological Characterization of Methiodone.

[B50] Wilde M. Sommer M. J. Auwärter V. Hermanns-Clausen M. (2020). Acute severe intoxication with cyclopropylfentanyl, a novel synthetic opioid. Toxicol. Lett. 320, 109–112. 10.1016/j.toxlet.2019.11.025 31778775

[B51] Wrbas S. Sundermann T. R. Auwärter V. Huppertz L. M. (2025). A case of fatal intoxication with the novel synthetic opioid N-pyrrolidino protonitazene. Int. J. Leg. Med. 140, 763–768. 10.1007/s00414-025-03618-8 41107543 PMC12956980

[B52] Zschiesche A. Theofel N. Braukmüller S. Ehrlich E. Jasyk M. Methling M. (2025). Deadly confusion of novel psychoactive substances: fatal outcome of ADB-BUTINACA mislabelled as 3’,4’-methylenedioxy-α-pyrrolidinohexiophenone. Forensic Toxicol. 44, 257–270. 10.1007/s11419-025-00746-z 41307856 PMC12858572

[B53] Zschiesche A. Schneider B. Nordhorn I. Baessmann C. Huppertz L. M. Kempf J. (2026). A novel workflow for fast elucidation of drug metabolites for screening – combining *in silico* metabolite prediction with trapped ion mobility QTOF-MS. Drug Test. Anal. 18, 841–851. 10.1002/dta.70062 42021506 PMC13327197

