# Supplementary material for: Case Report: Mislabeling of an online “alprazolam triazolobenzophenone” pill leads to severe intoxication with the novel synthetic opioid cychlorphine
Source: Front Toxicol. 2026 Oct 21;8:1951240. doi: 10.3389/ftox.2026.1951240 (PMC13730470; doi:10.3389/ftox.2026.1951240)
Supplement: Supplementary file 1 [file Table1.pdf]

Case Report: Mislabeling of an online "alprazolam triazolobenzophenone" pill leads to severe intoxication with the novel synthetic opioid cychlorphine

Annette Zschiesche[1,2], Johannes Nadler[3], Benedikt Pulver[1], Martin Scheu[1,2], Volker Auwärter[1*] Maren Hermanns-Clausen[3]

[1] Institute of Forensic Medicine, Forensic Toxicology, Medical Center – University of Freiburg, Faculty of Medicine, University of Freiburg, Freiburg, Germany

[2] Hermann Staudinger Graduate School, University of Freiburg, Freiburg 79104, Germany

[3] Poisons Information Center Freiburg, Department of General Pediatrics, Adolescent Medicine and Neonatology, Center for Pediatrics, Medical Center – University of Freiburg, Faculty of Medicine, Freiburg, Germany

*** Correspondence:**
Volker Auwärter
volker.auwaerter@uniklinik-freiburg.de

GC-EI-MS

One of the tablets was crushed, and 0.98 mg were dissolved in 1 mL of methanol. An aliquot of 100 µL was evaporated to dryness after addition of 50 µL isopropanol/HCl solution. The residue was derivatized with 100 µL acetic anhydride and 70 µL pyridine using microwave irradiation (7 min, 450 W). Then, it was mixed with 10 µL of internal standards, evaporated to dryness and reconstituted in 100 µL ethyl acetate. GC-EI-MS analysis was performed using an Agilent 6890 GC with an HP-5MS column (30 m × 0.25 mm × 0.25 µm), coupled to a 5973 mass selective detector and 7683 B injector. The method used was published elsewhere (Moosmann et al., 2012). One microliter of the sample was injected in splitless mode.

LC-DAD

The HPLC-DAD system consisted of a Nexera XR HPLC system and a SPD-M20A diode array detector (Shimadzu, Duisburg, Germany). Chromatographic separation was performed on a Kinetex® 2.6 µm C_{18} 100 Å, 100 × 2.1 mm (Phenomenex Ltd., Aschaffenburg, Germany) and a corresponding guard-column. A flow rate of 0.5 ml/min and the following gradient elution was applied: starting with 5% of solvent B, held for 1 min, increased to 45% within 9 min and to 98% within another 0.1 min, held for 1.9 min. The initial conditions were then restored within 0.5 min and held for 2.5 min to re-equilibrate the system. DAD-UV spectra were recorded from 200 – 400 nm, and the chromatogram (detection wavelength 280 nm) was used for semi-quantification of cychlorphine. Further method details are published elsewhere (Giorgetti et al., 2022).

Eluent A consisted of 1% acetonitrile, 1% ammonium formate (2 mM), and 0.1% formic acid in deionized water. Eluent B was prepared by mixing acetonitrile with 1% ammonium formate (2 mM) and 0.1% formic acid.

References:

Giorgetti, A., Brunetti, P., Pelotti, S., Auwärter, V., 2022. Detection of AP-237 and synthetic cannabinoids on an infused letter sent to a German prisoner. Drug Test. Anal. 14, 1779–1784. https://doi.org/10.1002/dta.3351

Moosmann, B., Kneisel, S., Girreser, U., Brecht, V., Westphal, F., Auwärter, V., 2012. Separation and structural characterization of the synthetic cannabinoids JWH-412 and 1-[(5-fluoropentyl)-1H-indol-3yl]-(4-methylnaphthalen-1-yl)methanone using GC–MS, NMR analysis and a flash chromatography system. Forensic Sci. Int. 220, e17–e22. https://doi.org/10.1016/j.forsciint.2011.12.010

Table S1: Additional substances detected in urine via general unknown screening with LC-QToF-MS.

**Analyte**	**LC-qToF-MS**
4-Acetylaminoantipyrine	detected
4-Aminoantipyrine	detected
4-Formylaminoantipyrine	detected
Caffeine	detected
…Theobromine	detected
…Theophylline	detected
Lidocaine	detected
Nicotine	detected
Cotinine	detected
…Hydroxycotinine	detected
Phenazone	detected
Rocuronium	detected
Xylometazoline	detected
